# Fellow-Workers and the Roll of Honour

**Published:** 1915-07-10

**Authors:** 


### ROUND THE WORLD.

[The Editor invites Early Information and Contributions Marked "Fellow-Workers."]

### Fellow-Workers and the Roll of Honour.

THE staff of consultants appointed to serve with our forces in the Mediterranean consists of a well-known physician specialising in nervous disorders, and five eminent London surgeons. These distinguished consulting physicians and surgeons, who have been given the rank of colonel in the R.A.M.C., are Dr. Purves Stewart, Mr. C. A. Ballance, Mr. V. W. Low, Mr. A. W. Mayo Robson, Mr. Charters J. Symonds, and Mr. A. H. Tubby.

COLONEL PURVES STEWART, M.D. Edin., F.R.C.P. Lond., has achieved fame by his brilliant observations on the diagnosis and treatment of diseases of the nervous system, his book on the "Diagnosis of Nervous Diseases" being widely read. He served in the South African War as physician to the Imperial Yeomanry Field Hospital, and has since then practised in London as a consulting physician, holding the posts of physician to Westminster Hospital, to the West-End Hospital for Nervous Diseases, and to the Royal National Orthopædic Hospital. It is understood that Colonel Stewart will, for the time being at any rate, be stationed at Malta.

COLONEL CHARLES A. BALLANCE, M.V.O., is chief surgeon to the Metropolitan Police, and holds various important hospital appointments, including those of surgeon to St. Thomas's and consulting surgeon to the National Hospital for Paralysis and Epilepsy in Queen Square. He qualified in 1881 by taking the M.B. Lond., when he was awarded first-class honours in each subject; whilst in the same year he won the gold medal at the B.S. Lond. Subsequently he became an F.R.C.S. Eng., and on sitting for the M.S. Lond. was again awarded a gold medal. Colonel Ballance, who has done important work in connection with brain and nerve surgery, will be on duty at Malta.

COLONEL VINCENT WARREN LOW, M.D., B.S. Lond., F.R.C.S. Eng., saw a good deal of military work in South Africa when he served as civil surgeon with the Field Force. A St. Mary's Hospital man, he was appointed to the staff of that institution not long after returning to London and resuming practice as an operating surgeon. For some years Colonel Low, who is now surgeon and lecturer on surgery at St. Mary's, was on the staff of the Great Northern Central Hospital, Holloway.

COLONEL A. W. MAYO ROBSON, C.V.O., F.R.C.S. Eng., is taking up duties in Egypt, where the Army surgeons will have the benefit of his extensive experience. Before retiring from active practice Colonel Mayo Robson was one of the most successful surgeons of his day, and secured for himself an exceptional position in the provinces before establishing himself in the front rank of London consulting surgeons. He was for many years on the staff of the General Infirmary at Leeds, and is, to-day, Emeritus Professor of Surgery in the University there.

THE promotion and work of Colonel Charters J. Symonds, M.S. Lond., F.R.C.S. Eng., were reported in these Notes quite recently (THE HOSPITAL, June 26, 1915). Not only Guy's men, but many other doctors in military service in the Mediterranean area will be glad to have the opportunity of turning for advice to this distinguished and very practical surgeon.

COLONEL ALFRED HERBERT TUBBY, M.S. Lond., F.R.C.S. Eng., whilst to-day being famous throughout the civilised world as an authority on orthopædic surgery, can look back to a brilliant student career. At the M.B. Lond. (1887) he won the gold medal in medicine, with honours in forensic medicine, and at the B.S. Lond. (1887) he also carried off the gold medal. His appointments include those of surgeon to Westminster Hospital and to the Royal National Orthopædic Hospital.

DR. LIONEL H. BOOTH, who, since the outbreak of war, has been on service with the Cameroons Expedition, has just been awarded a bronze medal by the Royal Humane Society. The gallant act thus recognised occurred last year in Southern Nigeria, when Dr. Booth rescued a native assistant from a deep river infested by alligators, bringing the unfortunate man safely to shore in spite of grave dangers and difficulties. A son of Dr. Lionel Booth, of Worthing, this brave member of the West African Medical Staff is an old Charing Cross Hospital man; after qualification he spent about two years as assistant surgeon in the Bahamas General Hospital, before going to the Dark Continent.

A ST. MARY'S man, Surgeon Frederick Whitby Quirke, R.N., went down with the *Princess Irene* when that vessel was blown up recently. Qualifying L.R.C.P., M.R.C.S., in 1908, Mr. Quirke was well known in his student days as a Rugby football player. Announcing the sudden and premature end to his career, the *St. Mary's Hospital Gazette* says: "He was full of life and energy, and was well liked by his fellow-students, who will all deplore his loss."

THE *St. Mary's Hospital Gazette* also reminds us that the late Captain William Frederic Higginson, who was killed during the dramatic landing in the Gallipoli Peninsula, was also at one time a pupil of St. Mary's Hospital Medical School. Entering as a student in 1897, he gave up reading when war broke out in South Africa, and accepted a commission in the Dublin Fusiliers. Subsequently prospering in his military career, he became in time captain and adjutant.

MR. HAROLD BURROWS, of Southsea, has gone to France to serve with one of the base hospitals, and whilst so connected will hold the rank of captain in the R.A.M.C. Formerly on the staff of the Seamen's Hospital at Greenwich, and the Bolingbroke Hospital, Wandsworth, Captain Burrows qualified from "Bart.'s" in 1899, and subsequently took the M.B., B.S. degrees of London University, and became an F.R.C.S. Eng. He is the author of a work on "Surgical Instruments and Appliances," which has run into a third edition, and at present holds the post of assistant surgeon to the Royal Portsmouth Hospital.

MR. F. H. REES, of Cardiff, who held a commission as temporary surgeon in the Royal Navy, is reported to have died of wounds received during the Dardanelles engagements. A student of Cardiff Intermediate School and University College Hospital, he qualified M.B., B.S. Lond. in 1912. In the early part of this year Mr. Rees took a commission in the R.A.M.C., but was subsequently transferred to the Naval Division. About three months ago he was ordered to the Mediterranean, where he appears to have been present during many fierce actions. He was a son of Dr. Alfred Rees, of Cardiff.

